# Photoelectrochromic Devices with Enhanced Power Conversion Efficiency

**DOI:** 10.3390/ma13112565

**Published:** 2020-06-04

**Authors:** Alexandros Dokouzis, Dimitra Zoi, George Leftheriotis

**Affiliations:** Renewable Energy Laboratory, Physics Department, University of Patras, 26500 Rion, Greece; phy5406@upnet.gr (D.Z.); glefther@physics.upatras.gr (G.L.)

**Keywords:** photoelectrochromics, iodine electrolyte, titanium oxide, tungsten oxide

## Abstract

In the present work, we propose a new architecture for partly covered photoelectrochromic devices with a modified anode layout, so that the TiO_2_ film is deposited first on the substrate, covering a small part of its surface, followed by the WO_3_ film that covers the remaining device area. As a result, the TiO_2_ film can be subjected to the proper thermal and chemical treatment without affecting the electrochromic performance of the WO_3_ film. The proposed design led to photoelectrochromic (PEC) devices with a power conversion efficiency (PCE) four times higher than that of typical partly covered devices, with a measured maximum of 4.9%. This, in turn, enabled a reduction in the total area covered by the photovoltaic unit of the devices by four times (to 5% from 20%), thus reducing its visual obstruction, without affecting the depth, uniformity and speed of coloration. A detailed study of the parameters affecting the performance of the new devices revealed that, with the cover ratio decreasing, PCE was increasing. The photocoloration efficiency also exhibited the same trend for cover ratio values below 15%. Storage of the devices in short circuit conditions was found to accelerate optical reversibility without affecting their photovoltaic and optical performance.

## 1. Introduction

Renewable energy production and energy savings are paramount for the sustainable development of mankind. Thus, smart windows and building-integrated photovoltaics have received considerable research interest in recent years [1,2], as they present promising tools towards the development of next-generation low-energy smart buildings [3].

A new emerging technology in this field is that of photoelectrochromic windows (PECs hereafter). They combine the functionalities mentioned above into one device [4]. More specifically, they consist of a solar cell (a dye-sensitized one in most cases, DSSC, hereafter) and an electrochromic device (EC, hereafter), with the electrical power required by the EC to change its transparency according to the environmental conditions provided by the Photovoltaic Cell (PV) element. PECs were reported for the first time in the seminal publication of Bechinger and coworkers [5], comprising, essentially, a DSSC with an ultra-thin TiO_2_ layer with low dye loading (for high transmittance) in the anode and an electrochromic WO_3_ film in the cathode, replacing the “standard” Pt catalyst. It was later realized that the device proposed therein could either exhibit fast coloration or fast bleaching, due to the competing processes of charge storage and charge loss at the WO_3_ layer. This design was superseded by the one proposed by Hauch and coworkers in [6], where both TiO_2_ and WO_3_ were placed in the anode, thus enabling coloration and bleaching to advance at similar speeds. However, even in that design, the low light absorption by the ultra-thin sensitized TiO_2_ resulted in solar cells with a low power conversion efficiency. This problem can be overcome by the “partly covered” design proposed by our research group in [7], which uses a thick, opaque TiO_2_ layer covering a low percentage of the device area (usually 10% to 20%), placed along the periphery of the device, out of the window optical path. Since then, many more combinations of materials and different architectures have occurred, [4] most of them being variations of the aforementioned designs. Recently, several advances have been proposed, such as partly covered PECs with polymer or gel electrolytes for improved stability [8,9], devices with sputtered TiO_2_ and WO_3_ layers [10], three-electrode devices (also called “photovoltachromics”) that de-couple EC and PV functionalities by appropriate cathode configurations [11,12], PEC devices using perovskite PV units [13,14] and finally, PECs with charge storage capabilities [15,16].

Regarding the exploitation of PECs in real life applications, it can be stated that they are still at an early stage, with several issues to be solved (such as stability in real operating conditions and performance enhancement) [4]. In the literature, many different designs have been presented. However, the optimum architecture and material combination has not been found yet. As a result, this field is open to research.

In the present work, in an effort to further improve the power conversion efficiency and the optical performance of the devices, we propose a new architecture for partly covered PECs. In the proposed architecture, the anode layout is changed, so that the TiO_2_ film is deposited first on the substrate, covering a small part of its surface, followed by the WO_3_ film that covers the remaining device area. As a result, the TiO_2_ film can be subjected to the proper thermal and chemical treatment that is described in numerous works regarding DSSCs [17,18,19,20,21,22,23,24,25,26,27] without affecting the performance of the EC film. The proposed design led to PEC devices with a PV efficiency four times higher than that of typical partly covered devices. This, in turn, enabled a reduction in the total area covered by the PV unit of the devices by four times (to 5% from 20%), thus reducing its visual obstruction, without affecting the depth, uniformity and speed of coloration. Furthermore, a detailed study of the parameters affecting the performance of the new devices is presented.

### 1.1. Layout and Operation of the Partly Covered PECs

A typical partly covered PEC architecture includes the following [9,28]:

A piece of glass with an electrically conductive transparent film such as SnO_2_:F (FTO, hereafter);

1. An electrochromic thin film (WO_3_ in this work), which is the active part of the device, as it changes its transparency;
2. A wide band gap mesoporous semiconductor film (TiO_2_ in this work) covering different percentages of the total surface area of the device. The film is sensitized with an appropriate dye (N719 in this work) in order to absorb a larger portion of the solar spectrum;
3. An electrolyte that contains a redox couple (usually I^−^/I_3_^−^) and lithium ions (Li^+^);
4. A second transparent FTO glass substrate with an ultra-thin platinum (Pt) coating.

The aforementioned parts 1, 3, 4 and 5 comprise the Photovoltaic Cell (PV) that provides the appropriate electrical potential to drive the Electrochromic Cell (EC), which is formed by parts 1, 2, 4, and 5.

Furthermore, electrochemically, parts 1, 2 and 3 form the anode and part 5 the cathode of the devices. Part 4, the electrolyte, is inserted between the anode and cathode.

**Figure 1:**
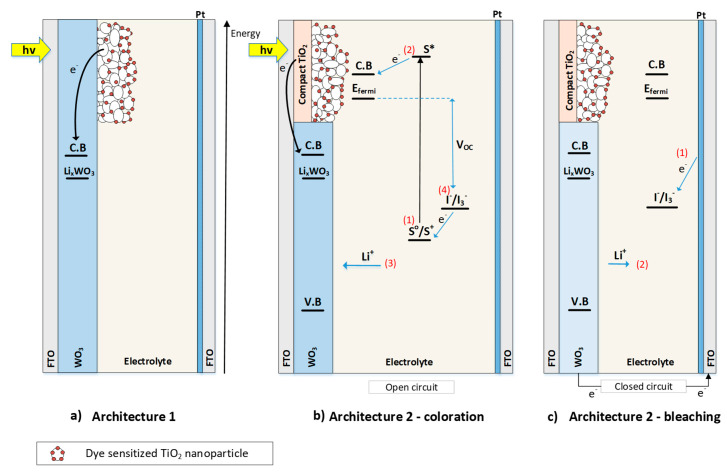
Scketches of “partly covered” photoelectrochromic (PEC) devices with different architectures. (**a**) Architecture 1, (**b**) coloration process—architecture 2. (**c**) Bleaching process—architecture 2.

In Figure 1 appear the different layouts of the partly covered PEC devices considered in this work. More specifically, the layout shown in Figure 1a is the typical design, used in our previous research [9,28,29,30] concerning PEC devices (to be called “architecture 1” hereafter). In that architecture, the WO_3_ film is deposited first on the FTO glass serving as the anode, and then the mesoporous TiO_2_ film is deposited on top of the WO_3_. In the new design proposed in the present work (to be called “architecture 2” hereafter), the order of film deposition is reversed; thus, the TiO_2_ film (consisting of a compact layer and the mesoporous film) is deposited first on the FTO, covering a portion of the substrate, while the growth of the WO_3_ film follows, covering the remaining area of the substrate, as shown in Figure 1b,c. With this architecture, the de-coupling of the deposition processes of the TiO_2_ and WO_3_ films has been achieved, allowing us to increase both the photovoltaic and coloration performance of PECs. Specific details regarding the fabrication of all the constituent layers are presented in Section 2.1.

The operation of these devices is as follows: incident light is absorbed by the dye (step 1—Figure 1b), electrons move to an excited state and are injected into the conduction band of TiO_2_ (step 2—Figure 1b). Next, the electrons are injected into WO_3_, either directly (architecture 1, Figure 1a), or through the FTO substrate, (architecture 2, Figure 1b). They then diffuse into the EC layer, which becomes negatively charged and Li ions intercalate into it for charge equilibration (step 3—Figure 1b). Thus, the WO_3_ film is colored.

The coloration takes place with the device in open circuit, meaning that the anode and the cathode are electrically separated. The following redox reaction occurs during coloration:

(1) $$xLi^{+}+xe^{-}+WO_{3}\left(bleached\right)\Leftrightarrow Li_{x}WO_{3}\;\left(colored\right)$$

Coloration, therefore, happens through the reduction in WO_3_ when the valence number changes from W^6+^ to W^5+^ (or W^4+^) by the intercalated Li ions (Li^+^) and electrons. (step 3—Figure 1b). The dye molecules are oxidized and their “regeneration” process (e.g., regaining the electron they lost) is carried out by the redox couple in the electrolyte (Step 4—Figure 1b) through the following reaction:

(2) $$2S^{+}+3I^{-}\Rightarrow 2S+I_{3}^{-}$$

In order to bleach the device, we short circuit the anode with the cathode. The energy level difference between Li_x_WO_3_ and I^−^/I_3_^−^ is such that a voltage is created between the two electrodes, and electrons spontaneously flow through the external circuit towards the cathode where they react with triiodide ions (step 1—Figure 1c). This reaction is catalyzed by the platinum layer and can be described as follows:

(3) $$I_{3}^{-}+2e^{-}\overset{\left(Pt\right)}{\Rightarrow }3I^{-}$$

Furthermore, oxidation of the tungsten oxide film results in the release of Li^+^ into the electrolyte with the simultaneous bleaching of the device in accordance with reaction (1).

## 2. Materials and Methods

### 2.1. Development of Materials

#### 2.1.1. FTO Substrates

Glass pieces with FTO coatings (sheet resistance of 16.7 Ω/sq, T = 80% in the visible range, trade name K-glass) 3 cm by 4 cm and 4-mm thick were used as substrates. Prior to deposition of any film they were cleansed as follows: (a) cleaning with a soft detergent, (b) ultrasound bath first in an acetone solution and then in a solution containing ethanol, both 5% v/v, (c) rinsing with deionized water and d) drying at 120 °C to remove the remaining moisture.

#### 2.1.2. TiO_2_ Compact Layer

In order to reduce photoelectron losses at the FTO/electrolyte interface, a compact TiO_2_ layer was deposited on top of the K-glass in the area where the mesoporous TiO_2_ would be deposited afterwards. A titanium disopropoxide bis(acetylacetonate) precursor solution (75% w/w in isopropanol) was used. It was further diluted in absolute ethanol (1 mL precursor in 5.4 mL EtOH). The deposition was carried out by spin coating (at 1000 rpm for 30 s) and the thickness of the films was below 100 nm. They were then dried at 120 °C for 5 min. Calcination at 400 °C or 500 °C was tried, with the former giving films with better blocking properties as those found by cyclic voltammetry testing, (see Section 3.1).

#### 2.1.3. Dye Sensitized TiO_2_ Films

For the precursor TiO_2_ paste, 2 g of TiO_2_ powder with Degussa P25 (80% anatase and 20% rutile) was placed in a porcelain mortar and was ground along with 0.45 mL of nitric acid solution (1.5 M). This was made so that the formation of large aggregates could be prevented [28,29]. Next, in order to dilute the paste and achieve the density value that was desired (1.6 g/mL), 2 mL of distilled water was added under continuous grinding. Lastly, a small quantity of Triton-X (5 drops) was added to the final solution in order to improve the adhesion of the paste on the FTO. The aforementioned procedure lasted for nearly 40 min and then the solution underwent ultrasonic treatment for 6–10 min in order to improve the homogeneity of the paste and to further break down any remaining aggregates.

**Figure 2:**
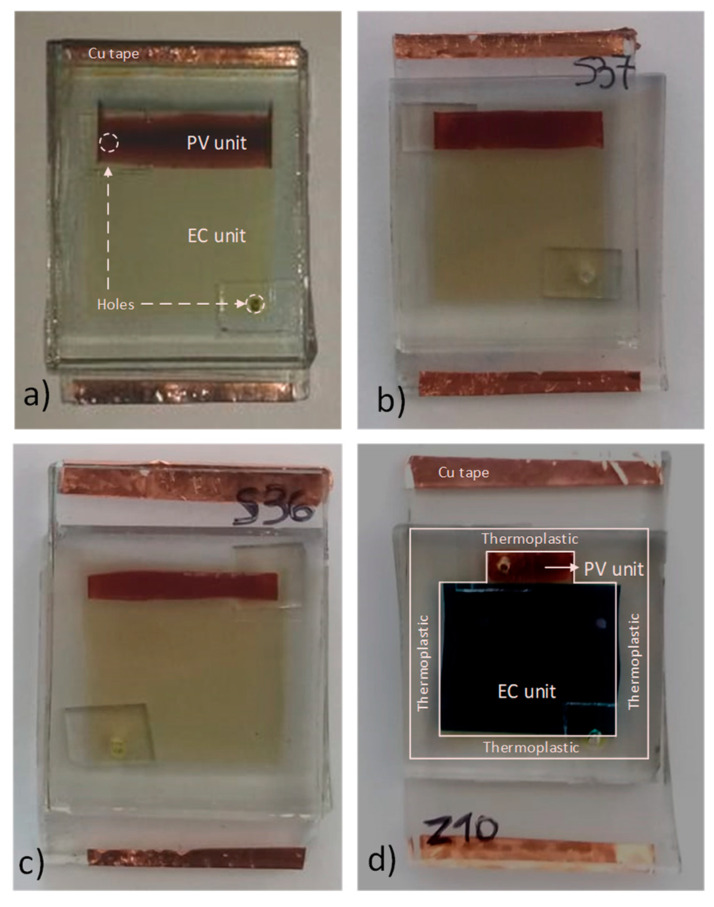
Digital pictures of “partly covered” PEC devices. (**a**) Bleached state—architecture 1, 20% cover ratio. (**b**) Bleached state—architecture 2, 16% cover ratio. (**c**) Bleached state—architecture 2, 12.5% cover ratio. (**d**) Colored state—architecture 2, 5% cover ratio.

TiO_2_ films were deposited using a doctor blade technique, with thickness of 10 μm ± 20%. The shape and dimensions of the films were specified each time with the use of a mask in order to cover a different fraction of the device area (e.g., 5%, 10%, 15%, etc., see also Figure 2). For this reason, the careful design of each PEC device prior to its construction was necessary.

The films were then air-dried and annealing of the substrates took place at 450 °C for 35 min in order to enhance the electrical conductivity of the TiO_2_ nanoparticles and also to burn away any organic materials that were still present from the cleaning process. Calcination at this temperature would not have been possible if the WO_3_ film had been deposited prior to the deposition of TiO_2_ due to re-crystallization of the WO_3_, which would have a direct impact on its electrochromic efficiency [30]. For that reason, in architecture 1, TiO_2_ films were annealed at 120 °C, exhibiting adverse effects in the power conversion efficiency of the fabricated PEC devices.

Sensitization of the films occurred with the use of a Ruthenium dye (Trade name N719 by Dyesol, GreatCell Solar, Queanbeyan, Australia). The samples were immersed for 24 h in a dye solution (0.3 mM in ethanol). Then, rinsing with methanol (MeOH) followed so that the excess dye was removed. It should be noted that sensitization took place after the deposition of WO_3_, which is described next.

#### 2.1.4. WO_3_ Films

WO_3_ was prepared by electron beam gun evaporation at room temperature in a vacuum chamber evacuated by a mechanical and turbo-molecular pump (P ≈ 10^−5^ mbar). The precursor was 99.99% pure WO_3_ powder. The thickness of the films was verified in situ by a quartz thickness controller. The deposited films were of an amorphous structure, and were highly transparent (≈ 74%) in the visible range [28,30] with an average thickness of 650 nm ± 10%. An appropriate mask was used to prevent the deposition of WO_3_ on the previously fabricated TiO_2_ film.

#### 2.1.5. Electrolyte

The electrolyte that was inserted in the devices of this work was prepared inside a glovebox, so that oxygen and moisture contamination was avoided. The composition of the electrolyte was as follows: 0.5 M lithium iodide (LiI), 0.005 M iodine (I_2_) and 0.5 M 4-tert-butylpyridine (4-TBP) dissolved in acetonitrile (ACN) [28].

#### 2.1.6. Counter Electrode

Firstly, we drilled two small holes, with diameters approximately of 1 mm each, to facilitate the pouring of the electrolyte. Next, the cleaning process of the substrate followed as described in Section 2.1.1. On the cleaned substrate, a platinum thin film was grown by an electrodeposition technique. The precursor solution was aqueous H_2_Pt_6_ (0.002 M) and the electrodeposition took place with the help of a three-electrode configuration. The equipment used was a Metrohm Autolab (Utrecht, The Netherlands) S.V (PGSTAT204) potensiostat. The FTO substrate was used as working electrode, with an Ag/AgCl electrode as reference and a Pt wire as the counter electrode. The deposition was carried out with a constant voltage equal to −400 mV imposed between the working and reference electrodes for a duration of 60 s. The as-prepared counter electrodes were of high transmittance (70%) in the visible range [28].

#### 2.1.7. Fabrication of the Devices

The fabricated PEC devices had dimensions of 3.0 cm × 4.0 cm and were of architecture 2, as described in Section 1.1. The anode and the cathode were placed so that their conductive surfaces faced each other. Furthermore, they were slightly displaced in order to preserve space for the electrical contacts, as seen in Figure 2. In order to effectively seal the apparatus, we used Syrlun (by Dyesol, with a thickness of 50 μm). The thermoplastic material was cut in the desirable shape and dimensions and was placed between the two electrodes. It served two purposes: (a) the definition of the device active area and (b) the creation of the space needed between the electrodes in order to insert the electrolyte afterwards (see Figure 2). Heating at 120 °C for 12–15 min followed in order to melt the thermoplastic. The pouring of the electrolyte was done through one hole, while the air leaked through the other. Finally, the two holes were sealed carefully, each with a small piece of thermoplastic and glass. To ensure better grip on the two electrical contacts, copper adhesive tape was used. The completed devices appear in Figure 2.

### 2.2. Characterization Methods

The cover ratio (CR) of each PEC device was calculated using the following formula:

(4) $$\mathrm{CR}=\frac{{\mathrm{TiO}}_{2}\;\mathrm{area}}{\mathrm{Total}\;\mathrm{area}}$$

where TiO_2_ area is the area that the titania film had been deposited on, and total area is the sum of the TiO_2_ and WO_3_ areas of the device.

#### 2.2.1. Optical Measurements

The optical behavior of each PEC device was studied in the visible range using a spectrophotometer (Perkin Elmer Lambda 650 UV/VIS, PerkinElmer, Inc, Waltham, MA, USA). The spectra of transmittance obtained from the measurements were used to calculate the luminous transmittance (T_lum_) according to Equation (5). It can be described as a spectral average, taking into consideration the human eye sensitivity f(λ) as a weighting factor. The spectra of transmittance T(λ) are the measurements obtained by the spectrophotometer.

(5) $${Τ}_{lum}=\frac{\int_{350nm}^{750nm}f\left(\lambda \right)\;\cdot \;Τ\left(\lambda \right)d\lambda }{\int_{350nm}^{750nm}f\left(\lambda \right)d\lambda }$$

The magnitude of the difference between the transmittance of the initial and colored state of the PEC device is referred as the variation in optical density and is given by:

(6) $${\Delta OD}_{\mathrm{col}}=\log\left(\frac{T_{\mathrm{lum},\;\mathrm{initial}}}{T_{\mathrm{lum},\;\mathrm{colored}}}\right)$$

where T_lum, initial_ and T_lum, colored_ are the luminous transmittance of the initial state and of the colored state, respectively. Deeper coloration of the device gives higher ΔOD values.

To calculate the coloration speed of PEC devices, photocoloration efficiency (PhCE hereafter) is an appropriate index, as has been proposed in previous works by our team [7,31], and has been adopted by the scientific community [4]. The PhCE curve is calculated only on the day of fabrication of the devices (day 0) according to Equation (7):

(7) $$\mathrm{PhCE}=\frac{{\Delta OD}_{\mathrm{col}}}{G_{T}\;\cdot \;t}\left[{\mathrm{cm}}^{2}\min^{-1}W^{-1}\right]$$

where G_T_ is the total power incident to the device (in W/cm^2^) and t is the exposure time (in minutes).

To calculate the PhCE values, on the day of their fabrication, the devices were subjected to the following measurements: (a) transmittance measurement for the initial (as prepared) transparent state, (b) exposure to the solar simulator described below at 1000 W/cm^2^ for time intervals of 0.5, 1, 1.5, 2, 3 and 6 min if needed and (c) measurement of the transmittance spectrum after each exposure interval.

The insolation of the devices was conducted with the use of a solar simulator (Oriel 96000 by Oriel Instruments, Stratford, CT, USA) fitted with an air mass filter (A.M 1.5G) to radiation equal to 1000 W/m^2^. The incident solar power was regulated with the help of a Si photoanode (VTB8440B) that was calibrated against a Broad Band Power Meter (Melles Griot 13PE001, Rochester, NY, USA) [30]. The devices were illuminated as shown in Figure 1.

#### 2.2.2. Electrical Characteristics

I–V curves were obtained with the use of the solar simulator and a Source Meter (Keithley 236, Keithley Instruments, Cleveland, OH, USA). The electrical efficiency of the P/V cell is given by [21]:

(8) $$n=\frac{V_{\mathrm{mp}}\cdot J_{\mathrm{mp}}}{G_{T}}$$

where the voltage and current density values that correspond to the point of maximum power are symbolized with V_mp_ and J_mp_, respectively, and the total incident power of G_T_.

Furthermore, in order to obtain a measurement of the PV quality, we used the fill factor (FF), which was calculated by [21]:

(9) $$\mathrm{FF}=\frac{V_{\mathrm{mp}}\cdot J_{\mathrm{mp}}}{V_{\mathrm{oc}}\cdot J_{\mathrm{sc}}}$$

where the open-circuit voltage and short-circuit current are abbreviated as V_OC_ and J_SC_ respectively.

The devices in our work were equipped with PV units of variable dimensions in order to meet the desirable cover ratio (CR) requirements in each case. I–V calculations were conducted with the use of a mask made of black material fitted with an opening that was used to calculate the current density. The devices were scanned from open-circuit to short-circuit conditions and no hysteresis was observed upon reversal of the scan direction. The device at the start of the I–V measurements was either fully bleached or fully colored. Between each successive data point, there was a 20 ms wait time.

#### 2.2.3. Extended Testing

The fabricated devices were stored in dark conditions, either at open or at short circuits, in their bleached state. Optical and I–V measurements were taken regularly the days that followed. A day of measurements included the following cycle: (a) optical and P/V tests for the initial state, (b) coloration of the device with the solar simulator for 6 min (1000 W/m^2^, A.M 1.5G), (c) optical and P/V tests for the colored state, (d) the bleaching of the device in the dark under SC conditions and e) optical tests when the device was fully bleached.

#### 2.2.4. Morphological and Structural Characterization

Scanning electron microscopy (SEM) images, as well as Dispersive Spectroscopy (EDS) scans of the film surface, were recorded using a JEOL (Tokyo, Japan) 6300 microscope at 5 kV.

A goniometer (Philips PW 1050/25) and a Cu Kα broad focus X-ray tube (λ = 1.5418 Å) was used to investigate the crystal structure of the compact TiO_2_ layers. The X-ray tube was operated at 40 kV × 30 mA from a Philips PW 11300/00/60 (Amsterdam, The Netherlands) extra stabilized generator. The scanning step was set to 0.02° and the range of the diffractograms was from 18° to 85°.

#### 2.2.5. Thickness Measurements

The thicknesses of the deposited films (WO_3_, mesoporous TiO_2_, compact TiO_2_) was measured ex situ with a stylus profilometer, Ambios XP−1, Ambios Technology, Santa Cruz, CA, USA. Pt was too thin to measure as it was several nanometers, which is lower than the minimum precision step height of the profilometer.

## 3. Results

### 3.1. Morphology, Structure and Electrochemical Properties of the TiO_2_ Compact Layer

**Figure 3:**
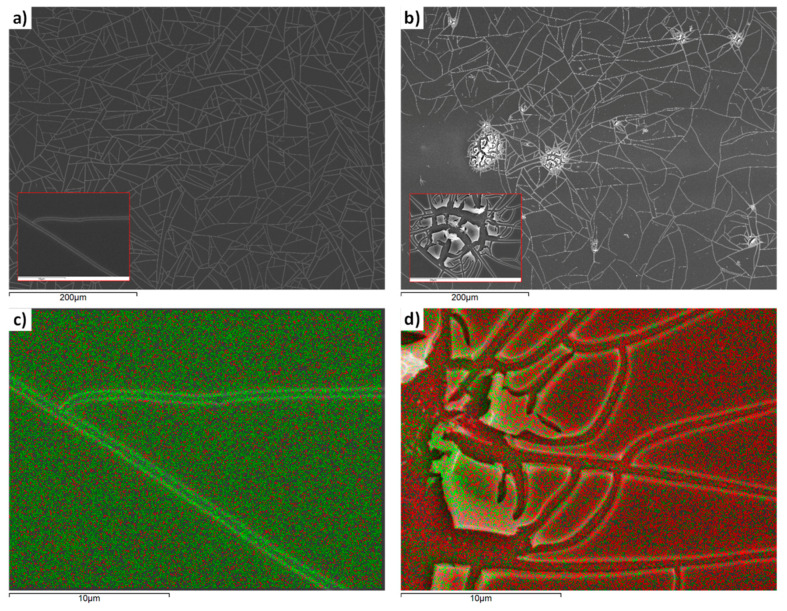
SEM images of thin films layers of compact TiO_2_ deposited on SnO_2_:F (FTO). (**a**) Annealed at 400 °C. (**b**) Annealed at 500 °C. (**c**) Annealed at 400 °C. EDS distribution of Sn (red) and Ti (green) at the film surface. (**d**) Annealed at 500 °C. EDS distribution of Sn (red) and Ti (green) at the film surface.

The compact TiO_2_ layers were prepared by spin coating as described in Section 2.1.2. Two different annealing temperatures were used, namely 400 °C and 500 °C, giving films with different properties. Indeed, as can be seen in the SEM image of Figure 3a, the film annealed at 400 °C appears uniform, apart from a network of cracks, probably caused by heating. 

**Figure 4:**
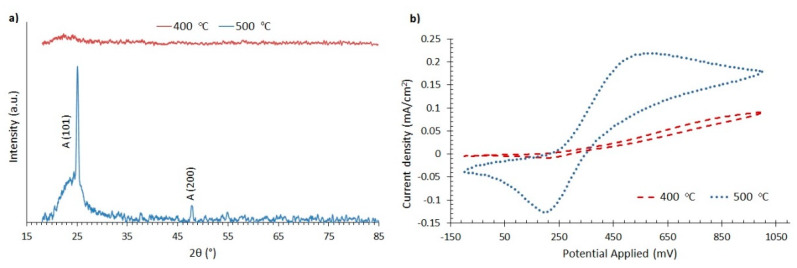
(**a**) XRD measurements of compact TiO_2_ films deposited on microscope slide glass. (**b**) Cyclic voltammetry measurements in an electrolyte with a ferrocene redox couple of compact TiO_2_ films on FTO annealed at 400 °C and at 500 °C.

These cracks, however, do not reveal much of the substrate, as can be seen in the EDS material analysis of Figure 3c. The film annealed at 500 °C also exhibits extensive cracking, as can be seen in Figure 3b, with regions where the FTO substrate is not covered by TiO_2_, as follows from the EDS material analysis shown in Figure 3d. XRD measurements, appearing in Figure 4a, reveal that the film annealed at 400 °C is amorphous, while that at 500 °C is crystalline with characteristic peaks of anatase [17].

In Figure 4b, cyclic voltammetry experiments are presented for compact TiO_2_ films on FTO, with the use of an electrolyte of the ferrocene redox couple (Fc/Fc^+^), consisting of 0.1 M Fc in acetonitrile. The (Fc/Fc^+^) couple was chosen because it is well known that it readily gives redox reactions on FTO [32], and thus it is a good electrochemical probe of the blocking effect of compact TiO_2_. The experimental setup also included a Metrohm Autolab S.V (PGSTAT204) potensiostat, an Ag/AgCl reference electrode and a Pt wire counter electrode. It is shown in Figure 4b that clear redox reactions between the working electrode and the electrolyte are realized only for the compact TiO_2_ annealed at 500 °C. This can be due to the exposed parts of FTO appearing in the SEM images (Figure 3b,d). On the other hand, Figure 4b reveals that the film annealed at 400 °C is an effective barrier, as it suppresses the redox peaks.

The morphological and structural characteristics of mesoporous TiO_2,_ evaporated WO_3_ and electrodeposited Pt films have been presented extensively in our previous work [8,17,33].

### 3.2. Optical and Electrical Characteristics

The devices of architecture 2 with different cover ratios were fabricated. Their optical and electrical performance were assessed and compared with those of devices from our previous work, based on architecture 1.

**Figure 5:**
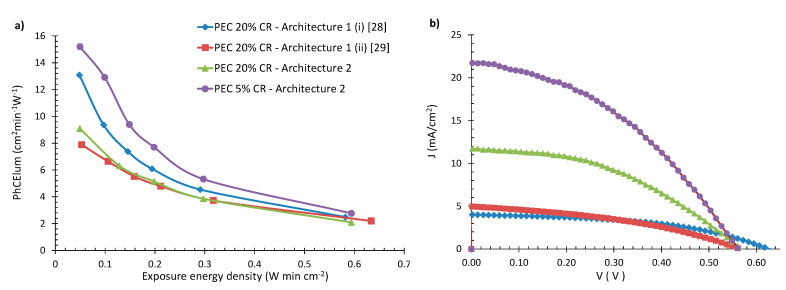
(**a**) Photocoloration efficiency vs exposure energy density for two PEC devices with architecture 1 and 20% cover ratio (CR) from our previous works, and two devices with architecture 2 and 20%, 5% CR and (**b**) J–V curves of these devices.

In Figure 5a, the photocoloration efficiency (PhCE) values versus the exposure energy density are presented for devices with different architectures. It can be observed that the highest coloration kinetics are exhibited for a device with 5% CR and architecture 2, followed by a device with 20% CR and architecture 1. Lower values of PhCE are shown for the device with 20% CR and architecture 2, along with one more device with 20% CR and architecture 1.

In Figure 5b, the J–V curves of the devices appearing in Figure 5a are shown. Their electrical characteristics are also presented in Table 1.

From those results, it follows that, among devices with the same cover ratio (e.g., 20%), those of architecture 2 exhibit a 2.4 times higher power conversion efficiency (PCE) compared to architecture 1. This increase in PCE occurs due to the proper treatment that devices with architecture 2 can be subjected to (discussed in detail in Section 2.1.2 and Section 2.1.3), which results in higher J_SC_ values. For the device with 5% CR (architecture 2), series and shunt resistance calculations gave R_sh_ equal to 1250 Ohms and R_s_ equal to 55 Ohms, and for the device with 20% CR (architecture 1), the values were R_sh_ = 1429 Ohms and R_s_ = 93 Ohms. It can be seen that architecture 1 gives higher series resistance than architecture 2, probably due to the WO_3_ film that intervenes between the TiO_2_ and the FTO.

The highest PCE value is achieved for a device of architecture 2 with 5% CR and is equal to 4.9%, more than four times higher than the best PCE obtained for architecture 1. Furthermore, the PhCE plots of Figure 5a show that this particular device exhibits the highest photocoloration efficiency, although the total area covered by its PV unit is reduced by four times (to 5% from 20%, see also Figure 2c). Compared to optimum DSSC solar cells fabricated in our laboratory with similar methods [34], the present devices exhibit a 32% lower PCE. This can be attributed to the limitations imposed on the electrolyte and the Pt layer in PECs, which need to exhibit high transmittance, only leaving room for the optimization of the TiO_2_ film.

**Figure 6:**
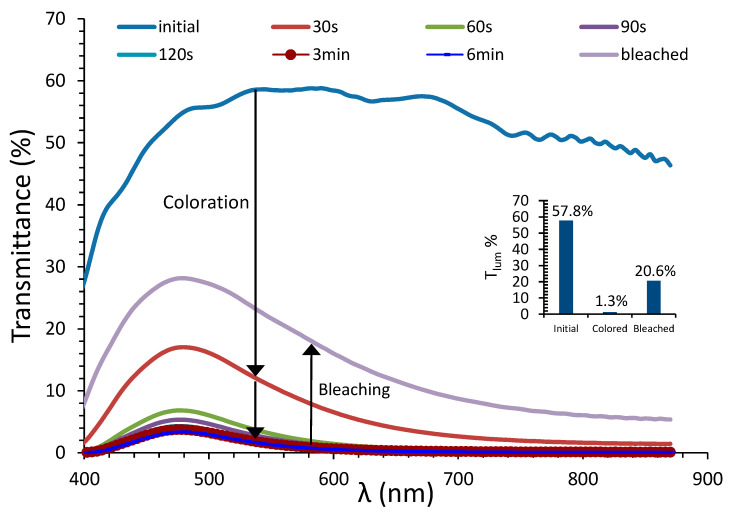
Transmittance spectra of various coloration stages as a function of the wavelength for the day of fabrication of a PEC device with architecture 2 (5% CR). Inset: luminous transmittance values for the initial, fully colored, bleached states of the device.

In Figure 6, the transmittance spectra of the device with architecture 2 and 5% CR are presented. 

The inset shows the luminous transmittance values for the corresponding initial, fully colored and bleached states of the PEC device. It follows that the optical behavior of the device is similar to that of devices with architecture 1, presented in our previous research [9,28,29,30], with a 44.5% contrast ratio between the initial and fully colored states, which is among the highest contrast ratio values presented in our previous work. Thus, the reduction in cover ratio does not appear to impede optical properties.

In the review of A. Cannavale and coworkers, [4], the optical performance of various PEC devices appearing in the literature is presented. The transmittance modulation (ΔΤ) as well as contrast ratio values of the devices presented in this work are classified in the highest half of the values presented therein.

It should also be noted that, on the day of fabrication, full bleaching cannot be achieved by short-circuiting the device for 6 min, as can be seen in Figure 6; therefore, the device is not fully reversible. This is a known feature of partly covered PECs [9,28,30]. This problem is rectified after a few days of storage, as the electrolyte gradually fills the pores of WO_3_. More details on device reversibility are presented in Section 3.3.1.

It can therefore be concluded that the implementation of architecture 2 on PEC devices brings about significant improvements regarding both their photovoltaic and optical performance.

### 3.3. Effects of the Cover Ratio

The results presented so far indicate that the cover ratio of the PEC devices affects their electrical and optical properties. In order to elucidate these effects, devices with various cover ratios ranging from 5% to 30% were fabricated and their properties are presented next.

**Figure 7:**
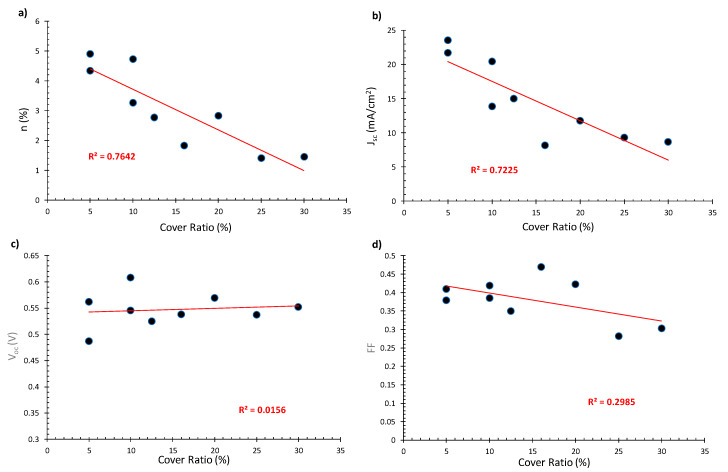
Electrical characteristics (day of fabrication) for devices of architecture 2 as a function of the cover ratio: (**a**) PCE, (**b**) J_SC_ (**c**) V_OC_, (**d**) fill factor (FF).

In Figure 7, the PCE, J_SC_, V_OC_ and FF values versus the cover ratio of the devices are presented. It can be observed that, with a decreasing CR, the PCE increases. 

This is mostly due to an increase in the short-circuit current density, as seen in Figure 7b. Regarding V_OC_ and FF values, it can be seen that they do not depend, to a large extent, on the cover ratio. The dispersion of experimental points observed in Figure 7 can be attributed to discrepancies in the characteristics of different devices. For example, there are considerable variations in the thickness of the TiO_2_ and WO_3_ films, caused by the deposition processes, as discussed in Section 2.1.3 and Section 2.1.4. The increase in PCE with decreasing cover ratio is probably an effect of device geometry and total area that is known to affect the performance of DSSCs [35].

In order to compare the coloration kinetics of various devices, the PhCE values of the first coloration step (e.g., 30 s or 0.05 W min cm^−2^ of exposure) were used. It is well known that the PhCE values of devices with different characteristics differ at the first coloration stages [30] and, for larger exposure times, they tend to converge [31], as shown in Figure 5a.

**Figure 8:**
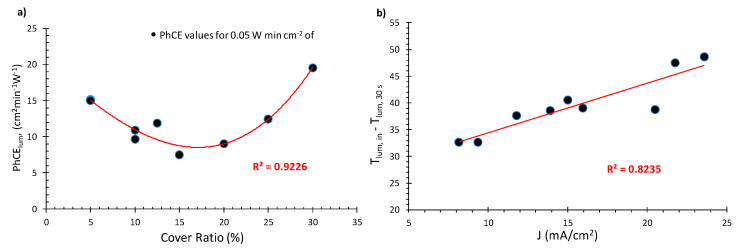
(**a**) Photocoloration efficiency values for 0.05 W min cm^−2^ of exposure. (**b**) T_lum,in_ – T_lum, 30 s_ as a function of SC current density for the day of fabrication.

In Figure 8a, the photocoloration values (PhCE) for 30 s of light exposure (0.05 W min cm^−2^) as a function of the CR are presented.

It can be seen that, starting at 30% and down to 15%, with CR decreasing, the values of the PhCE also decrease, which indicates that coloration speed also follows this behavior. This result is in agreement with our previous research [36], where it has been specified that this phenomenon could be attributed to the time that photoelectrons require to diffuse across the electrochromic unit. A lower area of coverage is linked with larger diffusion times and thus lower coloration speeds.

However, as the cover ratio decreases below 15%, an interesting phenomenon occurs: the PhCE values show an upward trend, reaching a maximum of approximately 15 cm^2^min^−1^W^−1^ for 5% CR. This probably occurs because, as seen in Figure 7a the PCE values increase with the decreasing cover ratio, due to the increase in the photocurrent density, as shown in Figure 7b. Thus, for cover ratio values below 15%, the factor with the highest significance regarding photocoloration efficiency appears to be the PCE of the PV unit, whereas above 15% would be the area ratio between the PV and the EC films.

As can be seen in Figure 8a, devices with 5% CR exhibit similar PhCE values as their 25% CR counterparts. However, one major advantage of the former is the fact that they exhibit higher PV performance and also present the lowest obstruction of the effective EC window among the other devices tested.

The device with 30% CR exhibits the highest PhCE value (20 cm^2^min^−1^W^−1^) compared to the other devices of architecture 2 that were tested and shown in Figure 8a. However, the high coverage area of the TiO_2_ increases the optical obstruction, which, in this case, is significant. Therefore, it is considered unsuitable for practical use.

Furthermore, in an effort to find a correlation between the optical and electrical characteristics of the devices, we compared various parameters against one another, such as PhCE, or ΔT_lum_ compared against I_SC_, J_SC_ η, V_OC_ and FF. The highest correlation was observed between ΔΤ_lum_ (e.g., T_lum,initial_ – T_lum, 30 s exposure_) versus J_SC_ with an R^2^ = 0.824, as shown in Figure 8b. It can be observed therein that the transmittance difference at the first stage of coloration is linearly dependent on the photocurrent density of the PV unit.

From the results presented above, it can be concluded that the PEC devices of architecture 2 with 5% CR can combine fast coloration, high PV performance and low optical obstruction by the PV unit. A comparison of the PCE values presented herein with data from the review work of A. Cannavale et al. [4] reveals that the efficiency values of this work belong in the higher half of the range.

#### 3.3.1. Extended Testing

**Figure 9:**
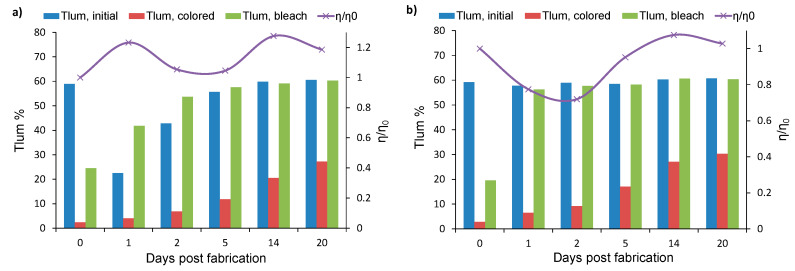
Luminous transmittance values and relative PCE (η/η_0_) vs days post fabrication of devices with 5% CR that were stored in (**a**) open circuit or (**b**) short circuit conditions.

In Figure 9, the luminous transmittance values for the initial, colored and bleached states and the relative PCE (η/η_0_) are presented for devices of architecture 2 with 5% CR. One device was stored in the dark in open circuit (OC) conditions and the other in short circuit (SC) conditions. Both devices remained in storage and were subjected to optical and electrical experiments at regular time intervals, as explained in Section 2.2.3.

It can be seen that the device stored in OC (Figure 9a) has a similar behavior as those of our previous works [28,30], which are devices of architecture 1 also stored in OC. Indeed, such devices undergo a transitional period with significant performance variations between consecutive days of testing, until they stabilize. Regarding optical reversibility, a device can be characterized as fully reversible if the T_lum, bleached_ is nearly equal to T_lum, initial_ on a single day of measurements. Reversibility usually occurs from 7 to 14 days post fabrication, as reported in our previous works [28,30]. In Figure 9a, the device stored in OC becomes reversible 14 days post fabrication when T_lum, initial_ is equal to T_lum, bleached_ with a value of 59%.

A considerable improvement can be observed in Figure 9b. Therein, the device stored in SC conditions exhibits a T_lum, initial_ value equal to 57% and T_lum, bleached_ equal to 56% on the first day of fabrication. As a result, reversibility occurs by only one day post fabrication, and the device remains reversible in the following days of testing. In both devices from Figure 9, variations in the PCE can be observed, which are due to changes in the electrolyte composition [9,28,30]. The PCE seems to stabilize to values just above the initial value η_0_. We can therefore conclude that storage of the devices in either OC or SC conditions does not seem to cause degradation in the DSSC.

It can be observed, in Figure 9, that T_lum,colored_ gradually increases with increasing storage time. This phenomenon can be attributed to loss reactions (Equation (3)) that take place at the WO_3_/electrolyte interface [28]. Losses hinder the WO_3_ coloration, and become more pronounced with time, due to the gradual wetting of the EC film by the electrolyte, as was found in our previous research [28]. An appropriate thin film barrier layer [9,28,29] can significantly suppress the losses and retain the deep coloration of the PEC devices. These barrier layers should meet some basic criteria: (a) They should be transparent in the visible range and (b) have a higher conduction band than that of the WO_3_ in order to prevent loss reactions at the WO_3_/electrolyte interface. One suitable material for barrier layers is zinc sulfide, which has been tested in our previous research with promising results [28,29]. Another possible material could be Al_2_O_3_.

Our future goal is to increase the efficiency (both electrical and optical) of PEC devices. This could be accomplished through the incorporation of the best features of our research, such as the new architecture, gel electrolytes and a suitable barrier layer into an optimized PEC device with enhanced performance and stability in real operating conditions.

## 4. Conclusions

- This work constitutes an experimental study of partly covered photoelectrochromic devices fabricated with a new architecture aimed to improve power conversion efficiency;
- In the new architecture 2, the TiO_2_ film is deposited first on the substrate, covering a small part of its surface, followed by the WO_3_ film that covers the remaining device area. This allows proper treatment of the TiO_2_ layer without affecting the morphology of the WO_3_, and thus the EC film retains its initial electrochromical capabilities;
- PEC devices of the new architecture 2 and 20% CR exhibit 2.4 times higher PCE than their architecture 1 counterparts. The highest PCE (namely 4.9%) was measured in a device of 5% CR and architecture 2, resulting in an overall increase in PCE between architecture 1 and 2 of 415%;
- The effect of cover ratio on PCE was evaluated. It was found that, with decreasing CR, the PCE values increase mostly due to the increase in photocurrent density;
- The effect of the cover ratio on the coloration speed of the devices was also tested. It was found that when starting at 30% CR, down to 15% with CR decreasing, the values of the PhCE (at 0.05 W min cm^−2^ of exposure) also decrease in accordance to our previous research. However, below 15% CR, an upward trend was observed, as devices with 5% CR exhibit similar PhCE values to their 25% CR counterparts;
- Devices with architecture 2 and 5% CR can combine fast coloration, high PCE and low optical obstruction;
- Storage of the devices in short circuit conditions was found to accelerate optical reversibility without affecting their photovoltaic and coloration performance.

## Figures and Tables

**Table 1:** Electrical characteristics of the devices presented in Figure 2.

PEC Architecture	CR (%)	J_m_ (mA/cm^2^)	V_m_ (mV)	J_SC_ (mA/cm^2^)	V_OC_ (mV)	FF	n (%)
1(i)	20	2.86	412	4.00	628	0.469	1.18
1(ii)	20	2.78	344	5.05	566	0.384	1.08
2 2	20 5	8.50 14.26	332 344	11.74 21.75	568 561	0.423 0.401	2.83 4.90
